# Quality of care and respect of human rights in mental health services in four West African countries: collaboration between the mental health leadership and advocacy programme and the World Health Organization QualityRights initiative

**DOI:** 10.1192/bjo.2021.1080

**Published:** 2022-01-25

**Authors:** Maria Francesca Moro, Lola Kola, Olawoye Fadahunsi, Edward Munda Jah, Humphrey Kofie, Dawda Samba, Sametta Thomas, Natalie Drew, Emeka Nwefoh, Soumitra Pathare, Julian Eaton, Michelle Funk, Oye Gureje

**Affiliations:** Department of Epidemiology, Columbia University Irving Medical Center, USA; WHO Collaborating Centre for Research and Training in Mental Health, Neurosciences and Substance Abuse, Department of Psychiatry, University of Ibadan, Nigeria; Mental Health Coalition, Sierra Leone; Mental Health Society of Ghana, Ghana; UN World Food Programme, The Gambia; Liberia Coalition of Mental Health Services, Liberia; Policy, Law and Human Rights, Department of Mental Health & Substance Use, World Health Organization, Switzerland; CBM Country Office, Nigeria; Centre for Mental Health Law and Policy, Indian Law Society, India; CBM Global Disability and Inclusion and Centre for Global Mental Health, London School of Tropical Medicine and Hygiene, UK; Policy, Law and Human Rights, Department of Mental Health & Substance Use, World Health Organization, Switzerland; WHO Collaborating Centre for Research and Training in Mental Health, Neurosciences and Substance Abuse, Department of Psychiatry, University of Ibadan, Nigeria

**Keywords:** World Health Organization QualityRights, human rights, psychiatric services, West Africa, United Nations Convention on the Rights of Persons with Disabilities

## Abstract

**Background:**

Although recent reports suggest that service users in West African psychiatric facilities are exposed to poor quality of care and human rights violations, evidence is lacking on the extent and profile of specific deficits in the services provided to persons with mental health conditions.

**Aims:**

To evaluate the quality of care and respect of human rights in psychiatric facilities in four West African countries, The Gambia, Ghana, Liberia and Sierra Leone, using the World Health Organization QualityRights Toolkit.

**Method:**

Trained research workers collected information through observation, review of records and interviews with service users, caregivers and staff. Independent panels of assessors used the information to assign scores to the criteria, standards and themes of the QualityRights Toolkit.

**Results:**

The study revealed significant gaps in these facilities. The rights to an adequate standard of living and to enjoyment of the highest attainable standard of health were poorly promoted. Adherence to the right to exercise legal capacity and the right to personal liberty and security was almost absent. Severe shortcomings in the promotion of the right to live independently and be included in the community were reported.

**Conclusions:**

Inadequate appreciation of service users’ rights, lack of basic approaches to protect them and the non-promotion of rights-based services in these facilities are major problems that need to be addressed. Although it recognises the resource constraints and need for more human and financial resources, the study also identifies critical areas and challenges that require significant changes at the facility level.

Despite mental health conditions accounting for a third of the global burden of disability,^1^ more than 70% of people in need of mental healthcare do not have access to good-quality services.^2^ Countries in West Africa face similar challenges.^3–8^ In these countries, the mental health budget ranges from 0.5% (The Gambia) to 1.3% (Ghana) of the total health budget.^7^ Community-based models of care are not present in many places, and the main providers of mental health services are psychiatric hospitals.^9^ Furthermore, mental healthcare is usually provided in major cities, which can be hours away from where people with mental health conditions live.^7^ In addition to the shortage of services, those that do exist often operate independently, with little coordination at the national or local level.^10^ Many people with mental health conditions receive care from faith-based and traditional healers,^11^ because of widespread beliefs about the supernatural causation of mental health conditions,^5,12^ as well as the scarcity of specialist mental health services.^13^ Nevertheless, some significant contributions to mental health services and capacity development have been made in the past decade or so, to fill this gap. An example is the Mental Health Leadership and Advocacy Program (mhLAP), which aims to build leadership and advocacy capacity and address country-specific mental health service development needs.^6^ Also, community health workers have been trained in some countries to provide mental healthcare^10,14^ and, in other countries, local civil society organisations (CSOs) have created mobile outreach teams to provide emergency and preventive mental healthcare to rural and urban populations.^15^ Furthermore, CSOs have supported the creation of peer support teams, which have become an important component of community mental health programmes.^16^

Despite these efforts, much work remains to be done to improve the quality of mental healthcare in the region. Recently, psychiatric facilities in a number of West African countries have come under scrutiny for poor quality of care and human rights violations.^17,18^ People receiving care in some psychiatric facilities in these countries experience poor physical infrastructure, overcrowding, inadequate food and administration of obsolete medications. Furthermore, chaining, seclusion and restraint are frequently used. In 2012, to address issues such as these, the World Health Organization (WHO) launched the QualityRights initiative,^19^ which aims to transform psychiatric services and promote the rights of persons with mental health conditions across the globe.^20^ Among the QualityRights instruments is the QualityRights Toolkit, developed to evaluate the quality of care and respect of human rights in mental health facilities. The QualityRights initiative follows the framework of the United Nations Convention on the Rights of Persons with Disabilities (CRPD),^21^ which aims to ‘promote, protect and ensure full and equal enjoyment of all human rights by all persons with disabilities’, including those with mental disabilities. When countries ratify the CRPD, they have a legal obligation to abide by its principles. Among the several West African countries that have ratified the CRPD are The Gambia, Ghana, Liberia and Sierra Leone. Thus, it is important to assess how these countries are implementing the CRPD in their public psychiatric services, given the violations that have been reported in this sector. To our knowledge, no previous studies have evaluated the alignment of psychiatric services with the CRPD in sub-Saharan Africa. The main objective of this study was to fill this gap by evaluating the quality of care and respect of human rights in psychiatric facilities in four Anglophone West African countries: The Gambia, Ghana, Liberia and Sierra Leone.

## Method

### Settings

The four West African countries were selected based on participation in the mhLAP initiative and willingness of the facilities in these countries to participate in the study. In The Gambia, the study was carried out in the Tanka-Tanka Psychiatric Hospital, located in the West Coast region. Tanka-Tanka is the country`s only psychiatric hospital, with a bed capacity of 100 and a staff strength of 58. In Ghana, the study was conducted in the Pantang Psychiatric Hospital, located in the Greater Accra region. Pantang is one of the three largest psychiatric hospitals in Ghana and serves approximately 8 million people. It is a 500-bed facility with in-patient and out-patient units, and employs 657 mental health workers. In Liberia, the study was carried out in the John F. Kennedy/E.S. Grant Mental Hospital, a national referral mental health hospital with a capacity of 80 beds. The facility has a staff strength of 46 and a small rehabilitation centre. In Sierra Leone, the study was conducted in the Sierra Leone Psychiatric Hospital. This facility is situated at the east end of Freetown in the Kissy community, and it is the only mental hospital in Sierra Leone, with a bed capacity of 150 and a staff strength of 79. All the participating facilities provide pharmacotherapeutic as well as simple psychosocial services. Further details on the facilities evaluated and the staff employed can be found in Supplementary Appendix 1 available at https://doi.org/10.1192/bjo.2021.1080.

### Instrument

**Fig. 1:**
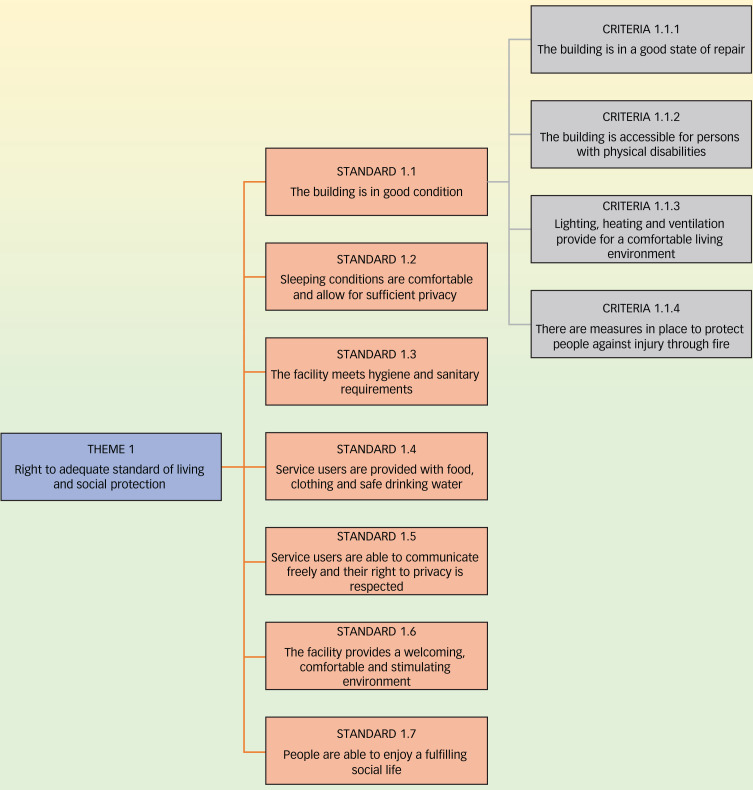
Example of the World Health Organization QualityRights Toolkit's organisation, with the division of themes into standards and criteria.

The quality of care and respect of human rights in psychiatric facilities were evaluated with the WHO QualityRights Toolkit,^20^ which adopts the human rights framework of the CRPD and includes five themes. Each theme focuses on specific CRPD rights: theme 1, the right to an adequate standard of living (Article 28); theme 2, the right to enjoyment of the highest attainable standard of physical and mental health (Article 25); theme 3, the right to exercise legal capacity and the right to personal liberty and security of person (Articles 12 and 14); theme 4, freedom from torture or cruel, inhuman or degrading treatment or punishment and from exploitation, violence and abuse (Articles 15 and 16); and theme 5, the right to live independently and be included in the community (Article 19). The themes are organised into standards, which consist of different criteria (see the example in Fig. 1). The QualityRights Toolkit has been previously used in other countries, such as India,^22^ Chile,^23^ the Czech Republic^24^ and Tunisia,^25^ to assess the quality of mental health services and is appropriate for the same purpose in West Africa.

### Assessments

In each country, the assessment of the selected psychiatric facility was conducted in three steps. In the first step, a QualityRights Coordinating Committee was established, including mental health professionals, human rights advocates, service users and other stakeholders. Then, the Committee selected a team of two/three research assistants based on their knowledge of, and capacity to understand, contemporary human rights and mental health issues. Among the research assistants selected were mental health professionals, human rights advocates and researchers with expertise on disability issues. In the second step, the team of research assistants collected information through (a) observation of the facilities and process of service delivery; (b) review of clinical and administrative records, including clinical records and nurses’ ward charts; and (c) interviews of selected service users, caregivers and staff. Research assistants took notes on the dedicated spaces in the WHO QualityRights Toolkit, and shot photos of the wards visited when they deemed it necessary.

In the third step, a panel of independent assessors conducted joint ratings, using the reports of the assessments provided by the research assistants. The panel of assessors was selected to ensure a broad range of skills and expertise and consisted of mental health professionals, service users, caregivers, legal practitioners, human rights advocates and representatives of CSOs working in mental health. No assessor worked or was otherwise involved in the provision of services in the facility assessed. Both the research assistants and members of the panel were trained in the use of the QualityRights Tools by country facilitators, who had previously participated in a 5-day training led by the project team and WHO consultants in Ibadan, Nigeria. Assessments by the research assistants were planned in collaboration with the management of each hospital.

### Analysis

The panel of assessors integrated qualitative and quantitative data through a mixed-methods convergent design to assign final ratings for each facility.^26,27^ Scores were assigned to the themes/standards/criteria through a process of detailed review of the reports of the assessments, discussions and consensus rating. Each theme, standard and criterion was scored as follows: ‘Not initiated-N/I,’ ‘Achievement initiated-A/I,’ ‘Achieved partially-A/P’ ‘Achieved in full-A/F’ and ‘Not applicable-N/A.’ Criteria were evaluated first. Then, based on the scores of the criteria, a score was assigned to the corresponding standard. Finally, scores of the standards were used to assign a score to each theme. When consensus among assessors could not be reached for a theme, standard or criterion, the lowest score was assigned. However, there was no report of significant disagreement in the scoring in any of the countries involved. Using the ratings provided by the panel, we have calculated the number of standards (with corresponding percentages) that received the different scores for each theme. The percentages were then used to create bar charts to represent the extent to which the specifications of the particular theme were achieved. Finally, we reviewed the qualitative descriptions and justifications provided by the panel in their reports to identify areas for improvement and provide recommendations. The same process has been previously used for analysing the data from assessments conducted in other countries.^22–25^

### Ethics

The authors assert that all procedures contributing to this work comply with the ethical standards of the relevant national and institutional committees on human experimentation and with the Helsinki Declaration of 1975, as revised in 2008. All procedures involving human participants were approved by the University of Ibadan/University College Hospital Ethics Committee (approval number UI/EC/13/0330) and the ethics committee of each participating hospital. All participants provided written informed consent.

## Results

Table 1 provides details on the number of interviews conducted in each facility. To ensure comprehensive information was obtained about the services provided in the facilities, at least eight people were interviewed in each stakeholder group. Following the WHO QualityRights Toolkit guidelines, interviewees were selected to have a good representation of people using or working at the facilities. For instance, service users were selected among people of different genders and with different diagnoses, who had been recently admitted or had been in the facilities for some time and were receiving care from different wards (both out-service users and in-patients). To ensure that service users' voices were adequately represented, a minimum of 14 service users were interviewed in each facility. Different categories of staff were also interviewed (e.g. nurses, doctors, social workers, psychologists, other health professionals and orderlies). Both staff members who had worked at the facility for some time and those recently employed were selected for the interviews. Potential interviewees who required urgent medical attention (e.g. evidence of profound confusion or agitation, high fever, injury) or were experiencing difficulties in their ability of concentration (e.g. because of the effects of sedating medication), as determined by the research assistants, during the process of obtaining the informed consent were excluded from the study. We did not provide more details on the numbers of the different categories interviewed in each stakeholders group to protect the anonymity and confidentiality of the interviewees.

**Table 1:** Profile of respondents for the interviews

Country	Staff	Service users	Caregivers (family members or friends)
The Gambia	8	15	8
Ghana	10	20	10
Liberia	8	14	8
Sierra Leone	8	14	8

### Theme 1: the right to an adequate standard of living

**Fig. 2:**
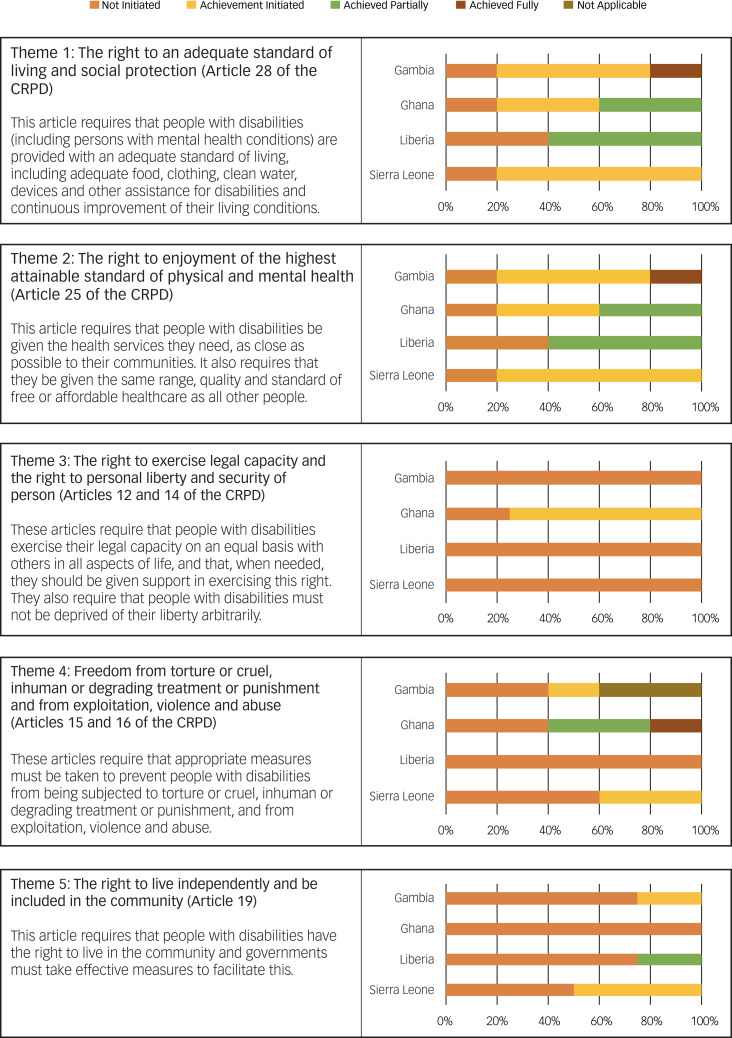
Adherence to the World Health Organization QualityRights Toolkit's themes (i.e. degree to which the rights of the CRPD are met) in the psychiatric facilities evaluated in the different countries. CRPD, United Nations Convention on the Rights of Persons with Disabilities.

Regarding overall adherence to theme 1, the hospital in Sierra Leone had actively initiated changes to ensure an adequate quality of living for service users, and the hospitals in the other countries had partially met the standards of this theme (see Fig. 2 and Supplementary Appendix 2).

In all countries, buildings were found suitable for use, although in need of renovations or maintenance (e.g. paint peeling off, floor tiles missing, insufficient lighting). Works had been undertaken to make the buildings more accessible (e.g. creating disability ramps, enlarging toilet cubicles); however, all hospitals presented barriers to access for people with disabilities. Although fire extinguishers were present in the facilities, staff and service users lacked knowledge on fire safety measures. Overall, service users’ sleeping conditions were comfortable, with separate wards for men and women in all hospitals. Clean beds and bedsheets were provided in all countries except for Ghana. However, patients were not allowed to lock their rooms for ‘safety reasons’, and had no individual lockers with locks; thus, their right to privacy was not fully respected. Service users had regular access to bathing and toilet facilities. These were generally clean at the time of the assessment, but, in all countries, they needed repairs (e.g. some taps were broken in The Gambia, some handles and seats were spoilt in Ghana, there was no hot water in Liberia). The evaluation teams also found shortcomings in the food served. In Liberia and Sierra Leone, the food was of good quality, but often not sufficient for service users. In The Gambia, the diet was unbalanced, whereas in Ghana, the quality of food varied depending on the ward examined. Sometimes, meals were served at the convenience of staff; for example, in Ghana, dinner was served too early because the kitchen staff wanted to close for the day. The hospitals respected service users’ right to wear clothes of their choice.

Service users were allowed to receive visitors during scheduled visiting times. Telephones were the only form of communication potentially accessible to service users. However, they were not always available. Also, sometimes staff were present during the communication, or there were restrictions in using these means of communication, thus limiting service users’ privacy. In Ghana and Sierra Leone, the evaluation teams found that the building environment was not stimulating and conducive to interaction. Service users had difficulties in remaining engaged in community life and activities. In The Gambia, some steps to promote interaction and participation were taken (e.g. creating a comfortable area for interactions between service users and visitors). In Liberia, social activities were organised within the facility compound, and service users sometimes participated in activities outside the facility (but only when requested by their families).

### Theme 2: the right to enjoyment of the highest attainable standard of physical and mental health

Adherence to theme 2 was partially achieved in Ghana, initiated in The Gambia and Sierra Leone, and not initiated in Liberia (see Fig. 2 and Supplementary Appendix 1).

Services were available, free of cost, to everyone who required treatment and support in The Gambia. In Ghana, all people, irrespective of origin or status, had access to treatment at the facility. However, service users with higher economic resources could pay for a better quality of care and had access to special wards. In Liberia, the facility had restrictions for admission based on age and ‘level of illness’ criteria.

All hospitals lacked adequate numbers of psychiatrists, although service users could access individual consultations with nurses. The number of professionals trained to provide psychosocial support and rehabilitation (e.g. occupational therapists, psychologists, social workers) was insufficient, so the prevalent treatment approach was pharmacological. Sometimes the facilities experienced shortages of medications (e.g. in Sierra Leone and Liberia) or there were not enough psychiatrists or medical doctors to review the prescriptions (e.g. in The Gambia, where nurse attendants prescribed medications although they were not licensed to do so).

Although service users had individual treatment plans in all facilities, these plans were solely based on mental health providers’ inputs from diagnosis and clinical observations. Service users were usually not provided the opportunity to express their preferences on treatment and recovery. Staff had little knowledge of international human rights standards, such as those included in the CRPD (except in Liberia, where some staff members had training on the CRPD). Social support mechanisms and networks to promote living independently in the community were not available in The Gambia, Liberia and Sierra Leone. In Ghana, staff facilitated linkages mostly between service users and other community mental health services. Some general health services were provided only in Ghana. Referral systems were in place in the other facilities. Some form of health education for service users was provided in Ghana (on mental health conditions, ‘adherence to medications’ and personal hygiene), Liberia and Sierra Leone (on hygiene and healthy habits). Reproductive health issues such as family planning and sexually transmitted diseases were not addressed in health education.

### Theme 3: the right to exercise legal capacity and the right to personal liberty and security of person

The psychiatric hospitals in The Gambia, Liberia and Sierra Leone had not initiated changes toward fulfilling service users' right to legal capacity and personal liberty and security. Only the facility in Ghana had taken initial steps toward fulfilling this theme (see Fig. 2 and Supplementary Appendix 1).

In none of the facilities were service users’ preferences on the place and form of treatment given a priority. Most of the time, service users were brought into the facility without their consent, by family members or police officers. Informed consent for admission and treatment was provided by family members, whereas service users had no voice on these matters since they were deemed ‘incapable of making decisions’. At other times, these decisions were taken by health providers without consulting family members. No procedures and safeguards were in place to prevent detention and treatment without free and informed consent. Even in countries where legal avenues to appeal detention and treatment existed, such as in Ghana, service users were not informed of this possibility. Overall, service users did not exercise their legal capacity nor were they given any support for exercising this right. Furthermore, the assessment teams found shortcomings in the right to confidentiality and access to personal health information. In The Gambia, service users indicated that they had no access to their personal information. Service users’ personal files were kept in the nursing section in an accessible cabinet without a lock, so that the right to confidentiality could have been easily infringed. In Ghana, Liberia and Sierra Leone, service users’ records were kept in a secure area under the custody of staff. However, service users were not informed of their right to access their records.

### Theme 4: freedom from torture or cruel, inhuman or degrading treatment or punishment and from exploitation, violence and abuse

The psychiatric hospitals in The Gambia, Liberia and Sierra Leone had not initiated changes toward fulfilling service users’ rights to freedom from torture or cruel, inhuman or degrading treatment or punishment and from exploitation, violence and abuse. Only the facility in Ghana had taken initial steps toward fulfilling these rights (see Fig. 2 and Supplementary Appendix 1).

The assessment teams found that verbal abuse by staff (e.g. shouting, having unfriendly reactions) and neglect were an issue in all facilities. These violations happened in particular when staff had a heavy workload because of the personnel shortage. Bullying among service users was also noted as an issue, and there were no measures in place for staff to address these situations. Seclusion and chemical and physical restraints were used in the facilities as a way of managing crises. Seclusion rooms were still used and were usually uncomfortable (e.g. they had bars and locks, no beds, mats or chairs). Furthermore, sometimes seclusion and restraint were used as a threat or form of punishment for service users who ‘misbehave’. Alternative methods to seclusion or restraint were rarely in place, and most of the staff had no training nor knowledge about these practices.

Electroconvulsive therapy was not practiced in any of the facilities evaluated (none owned the equipment necessary to perform the procedure). Medical and scientific experiments were not carried out in The Gambia, Liberia and Sierra Leone. In Ghana, medical and scientific experiments were conducted, but only upon approval of the ethical committee and with the informed consent of service users.

Safeguards were not in place to prevent ill treatment and abuse. In none of the facilities were there structured avenues for service users to lodge complaints, making it difficult to monitor and deal with cases of abuse. Service users could express their complaints informally, but they usually did not do it for fear of retaliation. Only in Ghana was there an independent body created by the government, whose mandate was to monitor the facility. However, the monitoring activities were inadequate.

### Theme 5: the right to live independently and be included in the community

Regarding theme 5 (see Fig. 2 and Supplementary Appendix 1), the psychiatric hospitals in The Gambia, Liberia and Sierra Leone had not initiated changes toward fulfilling service users’ right to live independently and be included in the community. Only the facility in Sierra Leone had taken initial steps toward fulfilling this right.

In all hospitals, service users often stayed longer than necessary because they had no resources for living independently and their families were not able or, sometimes, willing to provide support. Overall, because of financial constraints and the stigma toward people with mental health conditions in these countries, there are not many services in the community supporting people discharged from psychiatric hospitals in education, career development and employment opportunities. Even where these services exist, facilities were not able to link service users to them because of a lack of resources and logistics.

The hospitals did not prevent service users from participating in political, social or community life, but they did not actively support or promote it. Sometimes, service users were encouraged to participate in public activities outside the facilities upon approval from their families, and staff provided information on these activities.

## Discussion

This is the first evaluation of psychiatric services in West African countries, using the CRPD standards for the provision of care and treatment. Our study revealed significant gaps in the implementation of the CRPD principles in these countries. These findings align with those from studies using the WHO QualityRights Toolkit that were carried out in other countries.^22–25^

The right to an adequate standard of living (CRPD, Article 28) was not fully promoted because of the unsatisfactory state of most buildings and the lack of financial resources to make the necessary renovations and reparations. These findings reflect those of previous studies in West Africa.^3–8^ More resources need to be allocated by the governments to improve the facilities’ environment and make the buildings disability-friendly (e.g. ensuring that the entrances do not have stairs, the doors are wide enough to accommodate wheelchairs, there are hand-bars near toilets and bathtubs). Particular attention should be given to the provision of good quality food in sufficient quantities for the dietary needs of service users. Another priority is the installation of safety equipment (e.g. fire extinguishers at strategic locations), supported by the provision of training on safety measures for staff and service users.

The assessments also showed shortcomings in the achievement of the right to the enjoyment of the highest attainable standard of physical and mental health (CRPD, Article 25). The treatment provided in psychiatric facilities was mainly pharmacological, and shortages of medications were not uncommon. The number of staff was insufficient to meet service users’ care needs, which led to excessive workload and a decrease in the quality of care provided. Thus, alternatives to pharmacological treatments, such as psychosocial support and occupational therapy, were either not provided or inadequate. More efforts to train and hire a variety of professionals with different skills need to be made at the government and facility levels. In line with what was observed in other countries,^28^ the health workers in these facilities were generally not familiar with international human rights standards. Training on these issues, such as the WHO QualityRights online course, is now freely available and could help fill this gap if promoted within the facilities.

Adherence to the right to exercise legal capacity and the right to personal liberty and security (CRPD, Articles 12 and 14) was almost absent in all of the countries evaluated. These findings align with data from recent studies that show a strong endorsement of authoritarian and socially restrictive views toward people with mental health conditions in some West African countries.^12,29^ For instance, a study among Nigerian health professionals showed that providers hold negative attitudes toward people with mental health conditions and endorse paternalistic views.^30^ In this context, promoting the right to legal capacity and personal liberty and security may be challenging. However, steps in this direction need to be taken. It would be essential to educate service users (and health professionals) on their right to contribute to decisions regarding their recovery process, rehabilitation and reintegration into their communities. Supported decision-making mechanisms should be put in place, and service users should be educated to understand their right to nominate a representative to help them make decisions about treatment and other relevant issues. The introduction of peer support teams (workers or volunteers) in mental health facilities has been shown to have positive outcomes in empowering people with mental health conditions to enjoy their right to legal capacity,^22^ and could be a valuable resource in West African psychiatric services. Facilities should also develop guidance documents to manage the process and the manner in which informed consent is handled.

The rights to freedom from torture or cruel, inhuman or degrading treatment or punishment and from exploitation, violence and abuse (CRPD, Articles 15 and 16) were often violated. The assessment teams found evidence of verbal abuse against service users. Neglect was also an issue. Furthermore, seclusion and restraint were frequently used as a means to manage crises and, sometimes, as a threat and form of punishment. Health professionals rarely had training in de-escalation techniques, and service users-preferred intervention methods for crises were not taken into account. The CRPD requires States to implement alternatives to coercion and involuntary practices, and set goals and timelines for this change to happen in psychiatric facilities. Although some of the laws and policies in countries in West Africa are old and may not align with the CRPD on these rights, facilities can still do a lot to promote the rights of service users and stop abuses. For instance, they could provide training on alternatives to coercion and involuntary treatments (e.g. use of de-escalation techniques, advance directives, active listening and communications strategies, comfort rooms) for health providers and service users. All of these topics are covered in the WHO QualityRights in-person and online training. They could also develop internal policies setting out goals and deadlines for the implementation of these alternatives. Flyers and informative materials on the legal opportunities and appeal procedures should be made available to service users to enable them to appeal admission and detention effected without consent. It would also be crucial to set up structured complaints reporting mechanism. The reporting mechanisms should be independent of the facility (e.g. managed by the local CSOs or the government). When this is not possible, staff should be trained to properly handle the process without interfering with service users’ reports. This will provide an avenue for useful feedback on the standard of services delivered in the facility.

The present evaluation revealed shortcomings in the promotion of the right to live independently and be included in the community. There is a strong need for governments to create more community services, for facilities to liaise with the existing ones and for CSOs to create opportunities for service users after discharge. There are growing numbers of mental health CSOs (including organisations of people with mental health conditions) in The Gambia, Ghana, Liberia and Sierra Leone.^7^ These organisations provide peer support and are engaged in stigma reduction through advocacy, and could be valuable allies in promoting service users’ reintegration into their communities. The mhLAP has played a major role in helping to build the capacity of the members of some of these organisations, and could continue to play a role in the efforts of their leadership to raise community awareness and educate the public on issues around mental health.^31^ Mental health professionals and their organisations also have an essential role in promoting service users’ rights, including the right to live independently and be included in the community, and ensuring clinical practice is rights-based and dignity is preserved in the hospital setting. Therefore, they should collaborate with people with lived experience and mental health CSOs to achieve these objectives.^32^

The strengths of our study include the rigorously trained assessment teams, the use of a structured and comprehensive evaluation instrument and the inclusion of four countries from a region usually neglected in research. All of these factors contributed to a thorough examination of the psychiatric facilities. However, the study also has some limitations. Different assessment teams were employed in the four countries; thus, although they were supervised by the same central team in Nigeria, we cannot exclude that the differences in the scores were a result of subjectivity and specific cultural values of the local teams.

This study offers valuable baseline data on the quality of care and respect of human rights in psychiatric services in these countries. It identifies critical areas and challenges for improvement that facility managers and governments need to address in any effort designed to implement a human rights-based approach to mental health services in these countries, as well as in other low-resource settings. The underlying challenges that need to be addressed for any significant progress to be made include improved policy attention to mental health service, improved funding and staffing, and adequate training and retraining of staff. The latter is particularly important, given that providing care for the complex needs of people with mental health conditions, who are more likely to be discriminated against and exposed to human rights violations, is often more challenging than is commonly the case for many other health conditions.

## Data Availability

The data that support the findings of this study are available from the corresponding author, O.G., upon reasonable request.

## Supplementary material

For supplementary material accompanying this paper visit http://doi.org/10.1192/bjo.2021.1080. click here to view supplementary material
