# Quality of care and respect of human rights in mental health services in four West African countries: collaboration between the mental health leadership and advocacy programme and the World Health Organization QualityRights initiative – ADDENDUM

**DOI:** 10.1192/bjo.2022.67

**Published:** 2022-05-17

**Authors:** Maria Francesca Moro, Lola Kola, Olawoye Fadahunsi, Edward Munda Jah, Humphrey Kofie, Dawda Samba, Sametta Thomas, Natalie Drew, Emeka Nwefoh, Soumitra Pathare, Julian Eaton, Michelle Funk, Oye Gureje

**Keywords:** World Health Organization QualityRights, human rights, psychi-atric services, West Africa, United Nations Convention on the Rights of Persons with Disabilities

The above paper was published missed an acknowledgement regarding support from the Academic Freedom Fund. This has since been updated in the online PDF and HTML versions. The Publisher apologises for the omission.

## References

[ref1] Moro MF, Kola L, Fadahunsi O, Jah EM, Kofie H, Samba D, Thomas S, Drew N, Nwefoh E, Pathare S, Eaton J, Funk M, Gureje O. Quality of care and respect of human rights in mental health services in four West African countries: collaboration between the mental health leadership and advocacy programme and the World Health Organization QualityRights initiative. BJPsych Open 2022; 8(1), e31. doi: 10.1192/bjo.2021.1080. 35076357 PMC8811781

