# Supplementary material for: Quality of care and respect of human rights in mental health services in four West African countries: collaboration between the mental health leadership and advocacy programme and the World Health Organization QualityRights initiative
Source: BJPsych Open. 2022 Jan 25;8(1):e31. doi: 10.1192/bjo.2021.1080 (PMC8811781; doi:10.1192/bjo.2021.1080)
Supplement: Supplementary file 1 [file S2056472421010802sup001.docx]

**Supplementary Material 1. Description of the mental health facilities evaluated**

**The Gambia: Tanka-Tanka Psychiatric Hospital**

The Tanka Tanka Psychiatric Hospital of the Edward Francis Small Teaching Hospital (EFGTH) was established in 2009 as the country`s only psychiatric hospital with a bed capacity of 100. The hospital care consists primarily of providing psychotropic medications and some social, recreational, and occupational support services. The hospital has two adjacent wings (male and female) separated by burglar proofs, an admission room, consultation room, occupational therapy room, dining hall, staff room, and support units (laundry and kitchen). As the only psychiatric hospital in the Gambia, the hospital operates a community mental health service through a daily clinic in the Greater Banjul Area and has a mobile team that visits major rural facilities every three months. These visits have, however, been few and far between over the past few years.

The staff of the hospital consists of 58 members. A psychiatrist who comes twice a week, a trained child and adolescent psychiatrist who is stationed in the hospital daily. Four qualified psychiatric nurses (with bachelor’s degrees in general psychiatric nursing), a general nurse with three years of training in general nursing, a qualified social worker, two occupational therapists, and few nurse attendants who were trained at the EFSTH. The rest are auxiliary nurses, orderlies, and security.

**Ghana: Pantang Psychiatric Hospital**

The Pantang Psychiatric hospital is an offshoot of the Accra Psychiatric Hospital. It was declared open in 1975. The hospital is situated near a village called Pantang, about 1.6 kilometers off the Accra- Aburi road and 25 kilometers from Accra Central. The hospital was initially planned to be a Pan-African Mental Health Village. It is currently one of the largest psychiatric hospitals in Ghana. It has 500 beds, and service users are from over Ghana and nearby countries. It has in-patient and out-patient wards (for long- and short-term admissions). Pantang hospital provides pharmacotherapy and psychological counseling services. In line with the concept of offering integrated services, Pantang Psychiatric Hospital also provides reproductive and child health services and is equipped to deliver services at the maternal unit. It also has an eye clinic that does examinations and an oral health care service (Dental Unit).

The staff of the hospital consists of 657 members: 321 are professional nurses, 28 enrolled nurses, 11 community health nurses, 21 medical doctors (specialists and medical officers), 32 physician assistants, 3 clinical psychologists, 2 occupational therapists, 8 occupational therapists assistants, 44 health assistants, 7 laboratory technicians, 2 laboratory assistants, 1 radiographer, and 4 pharmacists. The rest are administrative staff members, security, and orderlies.

**Liberia: John F. Kennedy/es Grant Mental Hospital**

The JFK/ES Grant Mental Hospital serves an estimated population of about 4.5 million people. This facility stems from being a private mental health clinic owned by the late Dr. E. Samuel Grant, a psychiatrist, to a National Referral Mental Health Hospital and has a capacity of 80 beds. Before the civil war in Liberia in 1990, the hospital also had a rehabilitation center (the Catherine Mills Rehabilitation Center). This facility was originally located on a huge parcel of land and provided services to many people (including children and the elderly). The rehabilitation center was looted, and a portion of the land was taken away by squatters who built houses on it, leaving the ravaged rehabilitation center on a small parcel of land.

The staff of the hospital consists of 46 members. The facility has two psychiatrists, five social workers, two occupational therapist, seven mental health clinicians, eleven nurses, and other auxiliary staffs (e.g., kitchen staff and security staff).

**Sierra Leone: Sierra Leone Psychiatric Hospital**

The facility was the first psychiatric hospital in the Saharan Africa and was first named after the community (the Kissy Community) where it was located at its establishment in 1820. Sierra Leone Psychiatric Hospital is the only mental hospital in Sierra Leone. It has a capacity of 150-beds. It is situated at the East end of Freetown and was renamed in 2006 owing to the persistent stigmatization of both patients and staff by the public (it was previously known as Kissy Mental Hospital).

Until 2016, Sierra Leone relied on only one qualified psychiatrist. He recently retired after over 40 years of service and was succeeded by a new psychiatrist who is implementing the ‘Chain-Free Initiative’ across the country.

**Supplementary Material 2. List of standards scored to evaluate the quality of care**

**and respect of human rights in the psychiatric facilities**

**Theme 1: The right to an adequate standard of living and social protection (Article 28 of the CRPD).**

- Standard 1.1 The building is in good physical condition.
- Standard 1.2 The sleeping conditions of service users are comfortable and allow sufficient privacy.
- Standard 1.3 The facility meets hygiene and sanitary requirements.
- Standard 1.4 Service users are given food, safe drinking-water and clothing that meet their needs and preferences.
- Standard 1.5 Service users can communicate freely, and their right to privacy is respected.
- Standard 1.6 The facility provides a welcoming, comfortable, stimulating environment conducive to active participation and interaction.
- Standard 1.7 Service users can enjoy fulfilling social and personal lives and remain engaged in community life and activities.

**Theme 2: The right to enjoyment of the highest attainable standard of physical and mental health (Article 25 of the CRPD).**

- Standard 2.1 Facilities are available to everyone who requires treatment and support.
- Standard 2.2 The facility has skilled staff and provides good-quality mental health services.
- Standard 2.3 Treatment, psychosocial rehabilitation and links to support networks and other services are elements of a service user-driven recovery plan and contribute to a service user’s ability to live independently in the community.
- Standard 2.4 Psychotropic medication is available, affordable and used appropriately.
- Standard 2.5 Adequate services are available for general and reproductive health.

**Theme 5: The right to live independently and be included in the community (Article 19)**

- Standard 5.1 Service users are supported in gaining access to a place to live and have the financial resources necessary to live in the community.
- Standard 5.2 Service users can access education and employment opportunities.
- Standard 5.3 The right of service users to participate in political and public life and to exercise freedom of association is supported.
- Standard 5.4 Service users are supported in taking part in social, cultural, religious and leisure activities.

**Theme 4: Freedom from torture or cruel, inhuman or degrading treatment or punishment and from exploitation, violence and abuse (Articles 15 and 16 of the CRPD)**

- Standard 4.1 Service users have the right to be free from verbal, mental, physical and sexual abuse and physical and emotional neglect.
- Standard 4.2 Alternative methods are used in place of seclusion and restraint as means of de-escalating potential crises.
- Standard 4.3 Electroconvulsive therapy, psychosurgery and other medical procedures that may have permanent or irreversible effects, whether performed at the facility or referred to another facility, must not be abused and can be administered only with the free and informed consent of the service user.
- Standard 4.4 No service user is subjected to medical or scientific experimentation without his or her informed consent.
- Standard 4.5 Safeguards are in place to prevent torture or cruel, inhuman or degrading treatment and other forms of ill-treatment and abuse.

**Theme 3: The right to exercise legal capacity and the right to personal liberty and security of person (Articles 12 and 14 of the CRPD)**

- Standard 3.1 Service users’ preferences regarding the place and form of treatment are always a priority.
- Standard 3.2 Procedures and safeguards are in place to prevent detention and treatment without free and informed consent.
- Standard 3.3 Service users can exercise their legal capacity and are given the support they may require to exercise their legal capacity.
- Standard 3.4 Service users have the right to confidentiality and access to their personal health information.
